# Attenuation value of non-contrast computed tomography for spontaneous intracerebral hemorrhage: an important marker for the rapid identification of hematoma expansion

**DOI:** 10.3389/fneur.2026.1829603

**Published:** 2026-07-17

**Authors:** Jing Wang, Chuyue Wu, Jing Guo, Zhenjie Yang, Lei He, Shengli Chen, Lina Zhang

**Affiliations:** 1 Department of Neurology, Chongqing University Three Gorges Hospital, Chongqing, China; 2 School of Medicine, Chongqing University, Chongqing, China; 3 Chongqing Key Laboratory of Cerebrovascular Diseases, Chongqing University Three Gorges Hospital, Chongqing, China; 4 Chongqing Municipality Clinical Research Center for Geriatric Diseases, Chongqing University Three Gorges Hospital, Chongqing, China; 5 Department of Radiology, Chongqing University Three Gorges Hospital, Chongqing, China

**Keywords:** cerebrovascular diseases, hematoma expansion, nonenhanced computed tomography, relative CT attenuation value, spontaneous intracerebral hemorrhage

## Abstract

**Objective:**

Patients with spontaneous intracerebral hemorrhage (sICH) are highly prone to early hematoma expansion (HE). However, current limitations in the ability to quantify hematoma density restrict the prediction of HE risk. This study investigated whether relative CT attenuation value can predict HE in patients with sICH.

**Methods:**

This study included patients with sICH treated between January 1, 2020, and December 31, 2023. All patients underwent a baseline CT scan within 24 h of symptom onset and a follow-up CT scan within 36 h. The critical threshold for HE prediction based on the relative CT attenuation value was determined by plotting receiver operating characteristic (ROC) curves.

**Results:**

Of the 351 patients with sICH included in this study, 91 (25.9%) exhibited HE. After adjusting for confounding factors, multivariate logistic regression analysis indicated that the relative CT attenuation value could independently predict HE (*p* = 0.006). ROC analysis demonstrated that a relative CT attenuation value of 33.35 Hounsfield units (HUs) was the critical threshold for predicting HE, with an area under the ROC curve (AUC) of 0.665 (95% CI: 0.601–0.730). The sensitivity and specificity were 0.593 and 0.700, respectively. Of the 132 patients (37.6%) whose relative CT attenuation value was ≤33.35 HU, 54 (40.9%) exhibited HE. For predicting HE, the AUC, sensitivity, specificity, positive predictive value, negative predictive value, and accuracy of a relative CT attenuation value of ≤33.35 HU were 0.647 (95% CI: 0.589–0.705), 40.9, 83.1, 59.3, 70.0, and 67.2%, respectively.

**Conclusion:**

Relative CT attenuation value of hematoma can independently predict HE in patients with sICH.

## Introduction

Spontaneous intracerebral hemorrhage (sICH) is the second most common subtype of stroke, accounting for approximately 7–15% of all cerebrovascular events. It is associated with high mortality and morbidity rates, and overall patient prognosis is generally poor (1–4). Although few available therapeutic measures significantly improve long-term outcomes, early intensive intervention is crucial for acute management (5). A critical target during this window is HE, which usually occurs within the first 24 h after onset and represents a primary determinant of early neurological deterioration and subsequent adverse outcomes (6). Consequently, early identification of HE risk could facilitate timely and targeted interventions to prevent or reduce HE, potentially providing substantial clinical benefits in the treatment of cerebral hemorrhage.

Recent studies have demonstrated that qualitative non-contrast computed tomography (NCCT) markers, such as low-density areas, the black hole sign, the blend sign, and the swirl sign, are associated with HE in patients with sICH (7–10). However, these NCCT signs share similar morphological features, and there is a lack of consensus on appropriate terminology and diagnostic criteria, resulting in intra- and interobserver variability. In addition, radiology departments may use different CT scanner models, and even the same patient may exhibit significant differences in attenuation values when scanned by different CT machines. Furthermore, the attenuation values of hematomas can vary across different acquisition protocols or segmentation methods. Recently, researchers have introduced deep learning methods to calculate CT attenuation (11–13); however, these methods are not widely available in most hospitals and are time-consuming, which is not conducive to making early treatment decisions.

Therefore, a method that can quickly calculate the CT attenuation of hematomas in clinical settings and eliminate errors arising from differences in CT scanners, acquisition protocols, and segmentation methods is needed. Here, the difference between the CT attenuation on the hematoma side and the contralateral side was used to quantitatively describe the density differences in the hematoma, potentially reducing the errors. We hypothesized that this relative CT attenuation value can reliably predict early HE in patients with sICH.

## Methods

### Study design

This retrospective analysis utilized data from prospectively enrolled participants in an ongoing cohort at the Department of Neurology at Chongqing University Three Gorges Hospital. The study protocols was evaluated and approved by the Clinical Trial Ethics Committee of Chongqing University Three Gorges Hospital (Number: KS/2024063). Informed consent was obtained from all participants or their legally authorized representatives in accordance with the Declaration of Helsinki (National Medical Research Registration and archival information System: https://www.medicalresearch.org.cn. Unique identifier: MR-50-23-001346).

### Participant recruitment

This study included patients 18 years or older with sICH who were treated with minimally invasive surgery at Chongqing University Three Gorges Hospital between January 1, 2020, and December 31, 2023. Eligible patients also underwent a baseline NCCT scan within 24 h of symptom onset and a follow-up NCCT scan within 36 h of the initial scan. Patients were excluded if they had secondary ICH (due to cerebral aneurysms, Moyamoya disease, arteriovenous malformations, tumors, or hemorrhagic transformation of a cerebral infarction), primary intraventricular hemorrhage (IVH), or a baseline ICH volume of < 1 mL.

### Imaging analysis

**Figure 1:**
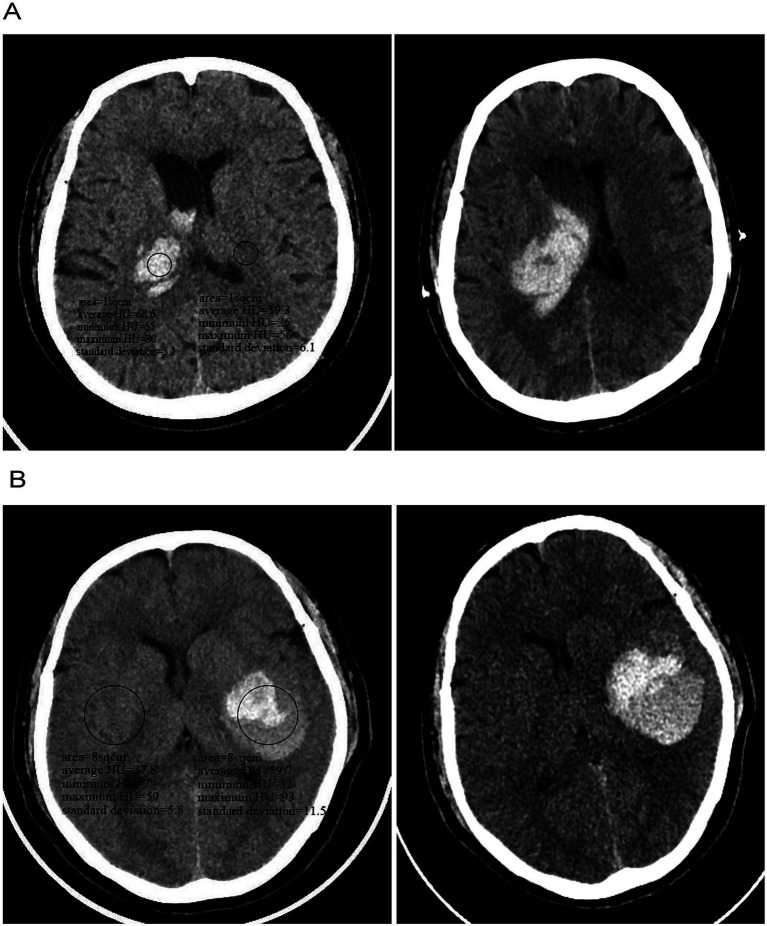
Two cases of patients with hemorrhage. **(A)** A 70-years-old female case with right thalamic hemorrhage. The onset time was 14:38 on Sep 1, and the time of arrival at the emergency department of our hospital was 17:10. The first NCCT scan was completed at 17:23. The CT attenuation of the largest region of interest on the side of the hematoma was 68.6 HU, and the CT attenuation of the area of greatest interest contralateral to the hematoma was 39.3HU. So, the relative CT attenuation value was 29.3 HU. Four hours later, the follow-up NCCT indicates the hematoma had expanded to 15.7 mL. **(B)** A 47-years-old male case with left basal ganglia cerebral hemorrhage. The onset time was 15:28 on Nov 29, and the time of arrival at the emergency department of our hospital was 18:00. The first NCCT scan was completed at 18:08. The CT attenuation of the largest region of interest on the side of the hematoma was 59.7 HU, and the CT attenuation of the area of greatest interest contralateral to the hematoma was 37.8 HU. The relative CT attenuation value was 21.9 HU. Twenty hours later, the follow-up NCCT indicates the hematoma had expanded to 44.2 mL. NCCT, non-enhanced computed tomography; HU, Hounsfield units.

NCCT examinations were performed using a multidetector CT scanner (REVOLUTION G CT or SIMENGES CT) with continuous axial slices of 2.4- or 2.5-mm thickness. The baseline hematoma volume was calculated using the Tada formula (ABC/2), and HE was defined as an absolute volume increase of > 6 mL or a relative increase exceeding 33% (14, 15). To determine the relative CT attenuation of the hematoma, we analyzed Digital Imaging and Communications in Medicine (DICOM)-formatted NCCT images stored in the picture archiving and communication system (PACS). Using the circular area tool in the PACS, we delineated a region of interest (ROI) within the largest cross-section of the hematoma. The ROI was sized to cover most of the hematoma while strictly avoiding the surrounding brain tissue. The contralateral ROI is selected as the brain region symmetrically corresponding to the hematoma’s ipsilateral ROI on axial imaging, primarily located at the basal ganglia-thalamus level, and reflects the average attenuation value of normal brain tissue at this level, corresponding to the anatomical position of the hematoma. The CT attenuation of the hematoma was then automatically calculated. Using the same ROI area, the CT attenuation of the normal brain tissue on the opposite side of the hematoma was also calculated. The difference between these two values represented the degree of hematoma attenuation on CT (Figure 1).

### Clinical data collection

The following clinical data of the enrolled patients were collected: sex, age, medical history of hypertension, diabetes, coronary heart disease, atrial fibrillation, anticoagulant/antiplatelet therapy, previous stroke, smoking, drinking, initially measured systolic blood pressure (SBP) and diastolic blood pressure (DBP), historical modified Rankin scale (mRS) score, poststroke mRS score, Glasgow Coma Scale (GCS), and National Institutes of Health Stroke Scale (NIHSS). We also recorded the hematoma volume on initial CT; hematoma volume on second CT; time from onset to initial CT; time from onset to second CT; presence of intraventricular hemorrhage; presence of subarachnoid hemorrhage; location of the hemorrhage; white blood cell (WBC) count; red blood cell count (RBC), platelet count (PLT), hemoglobin (HB), serum albumin (ALB), alanine aminotransferase (ALT), aspartate aminotransferase (AST), activated partial thromboplastin time (APTT), prothrombin time (PT), PT-International Normalized Ratio (PT-INR), thrombin time (TT), fibrin/fibrinogen degradation products (FDP), fibrinogen (FIB), total cholesterol, triglycerides, low-density lipoprotein (LDL), and high-density lipoprotein (HDL).

### Statistical analysis

All statistical analyses were conducted using R software (version 4.4.0). Continuous variables were presented as means [standard deviations (SDs)] or medians [interquartile ranges (IQRs)], as appropriate, and were compared using two-tailed Student’s *t*-tests or Mann–Whitney *U* tests. Categorical variables were expressed as frequencies and percentages and were compared using the *χ*^2^ test or Fisher’s exact test. A *p*-value of < 0.05 was considered statistically significant. Receiver operating characteristic (ROC) analysis was initially conducted to determine the critical threshold of the relative CT attenuation value for predicting HE. In addition, the sensitivity, specificity, positive predictive value (PPV), negative predictive value (NPV), and accuracy of the relative CT attenuation value for predicting HE were calculated. Univariate analysis was used to compare variables and identify potentially significant predictors of HE. The least absolute shrinkage and selection operator (LASSO) method was employed to screen for important variables associated with HE. Multivariate logistic regression was used to assess the independent association between the relative CT attenuation value and HE.

## Results

### Baseline characteristics

In total, 499 patients with sICH were initially evaluated, and 148 were excluded (76 due to lack of follow-up NCCT; 56 because the time to baseline NCCT exceeded 24 h; 5 due to reexamination CT time points exceeding 36 h; 6 with secondary ICH; 5 due to unobtainable imaging data). In total, 351 patients met the inclusion criteria (211 males and 140 females), of whom 91 (25.9%) experienced HE. The median age of the patients was 65 years ([IQR]: 55–72 years), with an age range of 30 to 93 years. The median baseline hematoma volume was 24.6 mL (IQR: 16.4–39.0 mL). The median time from symptom onset to the baseline CT scan was 3.6 h (IQR: 1.9–5.9 h). Baseline hematomas were in the basal ganglia (85.8%), cerebral lobes (9.7%), and infratentorial regions (brainstem, cerebellum) (4.5%). The patient’s baseline characteristics are shown in Table 1.

**Table 1:** Comparison of clinical and radiological characteristics of cohort patients.

Characteristics	No HE (260)	HE (91)	*p*
Age, years (median [IQR])	63.0 [54.0, 72.0]	68.0 [57.5, 72.0]	0.0887
Sex, female (%)	110 (42.3)	30 (33.0)	0.1494
SBP, mmHg (median [IQR])	168.0 [151.0, 185.0]	174.0 [162.5, 195.0]	0.0013
DBP, mmHg (median [IQR])	95.0 [84.0, 107.0]	98.0 [86.5, 108.5]	0.4828
HBP (%)	182 (70.0)	65 (71.4)	0.9017
DM (%)	17 (6.5)	9 (9.9)	0.4132
CHD (%)	16 (6.2)	10 (11.0)	0.1994
HF (%)	4 (1.5)	0 (0.0)	0.5761
Antithrombotic anticoagulant (%)	3 (1.2)	6 (6.6)	0.0111
History of intracerebral hemorrhage (%)	26 (10.0)	6 (6.6)	0.4019
History of cerebral infarction (%)	7 (2.7)	10 (11.0)	0.0039
Time from onset to NCCT, hours (median [IQR])	4.0 [2.2, 6.7]	2.6 [1.7, 3.7]	<0.0001
Time from onset to second NCCT, hours (median [IQR])	9.3 [6.7, 14.2]	6.9 [5.4, 10.8]	0.0001
MRS before the onset (median [IQR])	0.0 [0.0, 0.0]	0.0 [0.0, 0.0]	0.451
MRS after the onset (median [IQR])	4.0 [4.0, 5.0]	5.0 [4.0, 5.0]	0.3652
GCS (median [IQR])	10.5 [7.0, 13.0]	10.0 [7.0, 13.0]	0.4926
NIHSS (median [IQR])	16.5 [12.0, 21.0]	18.0 [13.0, 25.5]	0.0584
WBC, *10^9^/L (median [IQR])	10.1 [8.0, 12.9]	8.4 [6.0, 10.3]	<0.0001
RBC, *10^12^/L (median [IQR])	4.5 [4.1, 4.8]	4.6 [4.3, 5.0]	0.0445
HB, g/L (median [IQR])	137.0 [125.0, 148.0]	140.0 [130.5, 152.0]	0.0371
PLT, *10^9^/L (median [IQR])	191.5 [152.0, 225.2]	193.0 [153.5, 223.0]	0.9002
Urea, mmol/L (median [IQR])	5.2 [4.1, 6.5]	5.2 [4.2, 6.7]	0.5443
Creatinine, umol/L (median [IQR])	70.0 [57.0, 82.2]	71.0 [60.0, 90.0]	0.3089
Glucose, mmol/L (median [IQR])	7.2 [6.1, 8.6]	7.1 [5.9, 8.6]	0.4814
ALB, g/L (mean (SD))	43.6 (4.2)	44.3 (4.1)	0.1595
ALT, U/L (median [IQR])	16.7 [12.4, 24.6]	16.7 [11.6, 24.9]	0.8821
AST, U/L (median [IQR])	24.4 [19.7, 31.6]	24.4 [20.6, 29.0]	0.9254
PT, S (median [IQR])	10.8 [10.3, 11.3]	10.8 [10.3, 11.1]	0.902
INR (median [IQR])	0.9 [0.9, 1.0]	0.9 [0.9, 1.0]	0.6714
APTT, S (median [IQR])	24.1 [22.5, 25.4]	24.9 [23.4, 26.6]	0.0037
Fibrinogen, g/L (median [IQR])	2.8 [2.4, 3.4]	2.7 [2.3, 3.1]	0.0908
TT, S (median [IQR])	17.8 [17.1, 18.3]	17.9 [17.4, 18.8]	0.0063
FDP, mg/L (median [IQR])	1.4 [0.8, 2.7]	1.0 [0.6, 2.0]	0.0023
Cholesterol, mmol/L (median [IQR])	4.3 [4.0, 5.0]	4.3 [3.6, 4.9]	0.0148
Triglyceride, mmol/L (median [IQR])	1.0 [0.8, 1.3]	1.1 [0.9, 1.6]	0.0324
HDL, mmol/L (median [IQR])	1.4 [1.2, 1.6]	1.4 [1.1, 1.6]	0.1216
LDL, mmol/L (median [IQR])	2.5 [2.3, 3.1]	2.5 [1.9, 3.0]	0.0126
Baseline hematoma volume, mL (median [IQR])	25.1 [16.6, 38.1]	20.1 [12.8, 45.5]	0.4531
Re-examine hematoma volume, mL (median [IQR])	26.3 [16.9, 39.1]	40.0 [24.8, 69.8]	<0.0001
Location (%)			0.3111
Lobes	28 (10.8)	6 (6.6)	
Deep	218 (83.8)	83 (91.2)	
Cerebellum	11 (4.2)	1 (1.1)	
Brainstem	3 (1.2)	1 (1.1)	
Relative CT attenuation value, HU (median [IQR])	35.9 [32.2, 38.7]	32.3 [28.9, 35.8]	<0.0001

SBP, systolic blood pressure; DBP, diastolic blood pressure; HBP, high blood pressure; DM, diabetes; CHD, coronary heart disease; HF, atrial fibrillation; NCCT, non-enhanced computed tomography; mRS, modified Rankin Scale; GCS, Glasgow Coma Scale; NIHSS, National Institutes of Health Stroke Scale; WBC, white blood cell; RBC, red blood cell; PLT, platelet; HB, hemoglobin; ALB, serum albumin; ALT, alanine aminotransferase; AST, aspartate aminotransferase; APTT, activated partial thromboplastin time; PT, prothrombin time; PT-INR, PT-International Normalized Ratio; TT, thrombin time; FDP, fibrin/fibrinogen degradation products; FIB, fibrinogen; LDL, low-density lipoprotein; HDL, high-density lipoprotein; IQR, interquartile range; HE, hematoma expansion; HU, Hounsfield units.

### Critical value of the relative CT attenuation value determined via ROC analysis

**Figure 2:**
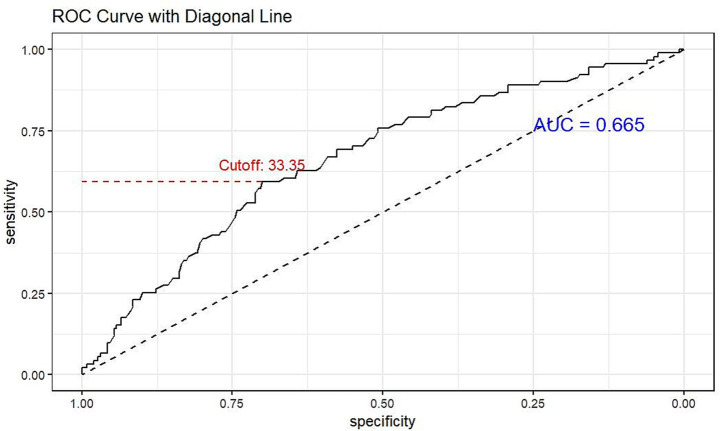
Receiver operating characteristic (ROC) curve of relative CT attenuation value for predicting HE.

**Figure 3:**
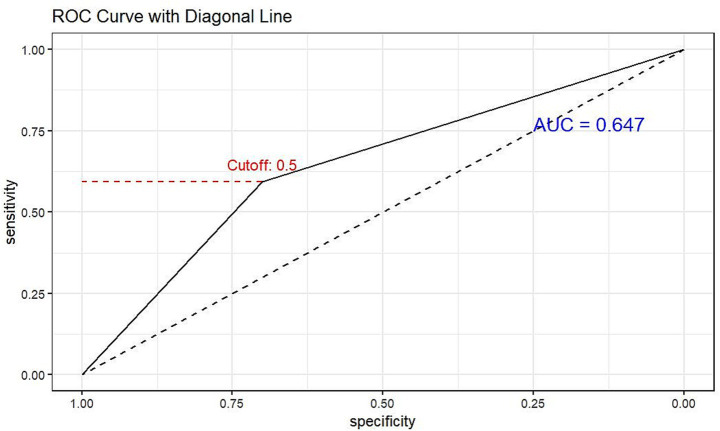
Receiver operating characteristic (ROC) curve of relative CT attenuation ≤33.5 HU for predicting HE.

We used the relative CT attenuation value of the hematomas and the corresponding HE outcomes from the cohort data to construct an ROC curve (Figure 2). A relative CT attenuation value of 33.35 Hounsfield units (HUs) was identified as the critical value for predicting HE, with an area under the ROC curve (AUC) of 0.665 and a 95% confidence interval (CI) of 0.601–0.730. The sensitivity and specificity were 0.593 and 0.700, respectively. Of the 132 patients (37.6%) with hematomas whose relative CT values were ≤33.35 HU, 54 (40.9%) experienced HE. The AUC, sensitivity, specificity, positive predictive value, negative predictive value, and accuracy of using a relative CT attenuation value ≤ 33.35 HU to predict HE were 0.647 (95% CI: 0.589–0.705), 40.9, 83.1, 59.3, 70.0, and 67.2%, respectively (Figure 3).

### Association between the relative CT attenuation value and HE

**Figure 4:**
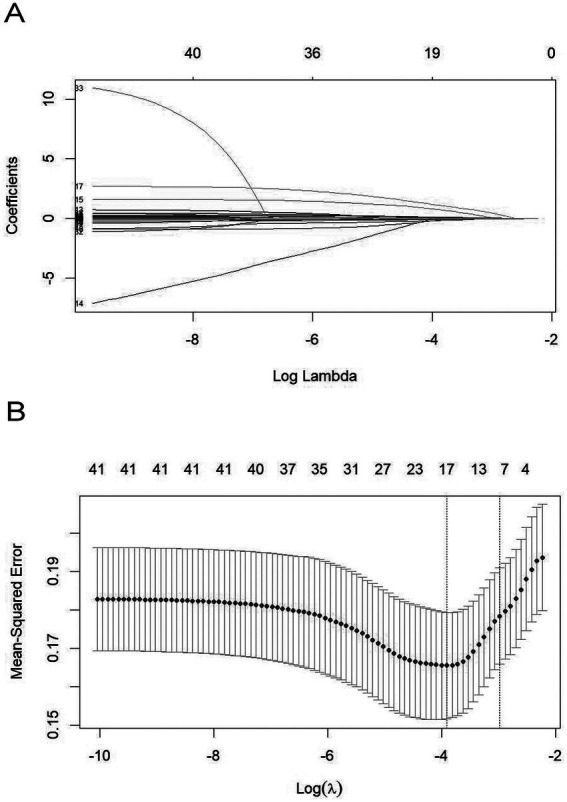
Variables selection by LASSO binary logistics regression model. **(A)** Each curve with different colors represents the change trajectory of each independent variable coefficient, the y-axis is the coefficient value; the upper x-axis is the number of non-zero coefficients in the LASSO model. **(B)** Represented the cross-validation result with different *λ* value, the left dot line represented lambda min which was the lowest *λ* of minimum mean cross-validated error, the right dot line represented the lambda.1se which was the largest value of *λ* such that error is within 1 standard error of the cross-validated errors for lambda.

Using LASSO regression, 13 independent variables with nonzero coefficient, including SBP; history of cerebral infarction; history of anticoagulation or antiplatelet therapy; time from onset to initial NCCT; time from onset to follow-up NCCT; NIHSS score; WBC count, Hb, LDL, and ALB levels; APTT; TT; and relative CT attenuation value (Figure 4), were selected for multivariate regression analysis. The results revealed that time from onset to NCCT, relative CT attenuation value, and WBC count were inversely associated with HE. History of cerebral infarction; NIHSS score; HB and ALB levels; and TT were positively associated with HE. Notably, the relative CT attenuation value independently predicted HE in patients with sICH [odds ratio (OR) = 0.92; 95% CI: 0.87–0.98; *p* = 0.006] (Table 2).

**Table 2:** Variables with statistical significance for predicting HE analyzed by multivariate logistic regression.

Variables	OR (univariable)	OR (multivariable)
SBP	1.01 (1.00–1.02, *p* = 0.007)	1.01 (1.00–1.02, *p* = 0.074)
History of cerebral infarction	4.46 (1.65–12.10, *p* = 0.003)	7.06 (1.84–27.09, *p* = 0.004)
Antithrombotic_anticoagulant	6.05 (1.48–24.71, *p* = 0.012)	3.81 (0.77–18.81, *p* = 0.101)
NIHSS	1.04 (1.00–1.07, *p* = 0.029)	1.07 (1.02–1.12, *p* = 0.003)
Time from onset to NCCT	0.82 (0.74–0.90, *p* < 0.001)	0.88 (0.78–0.99, *p* = 0.029)
Time from onset to second NCCT	0.92 (0.88–0.97, *p* = 0.001)	0.99 (0.93–1.04, *p* = 0.612)
WBC	0.84 (0.78–0.91, *p* < 0.001)	0.87 (0.79–0.96, *p* = 0.005)
HB	1.01 (1.00–1.03, *p* = 0.039)	1.02 (1.00–1.04, *p* = 0.040)
ALB	1.04 (0.98–1.11, *p* = 0.160)	1.10 (1.02–1.20, *p* = 0.020)
APTT	1.16 (1.06–1.26, *p* < 0.001)	1.22 (1.09–1.37, *p* < 0.001)
TT	1.32 (1.05–1.66, *p* = 0.017)	1.20 (0.90–1.61, *p* = 0.213)
LDL	0.62 (0.44–0.86, *p* = 0.004)	0.68 (0.46–1.01, *p* = 0.055)
Relative CT attenuation value	0.90 (0.86–0.95, *p* < 0.001)	0.92 (0.87–0.98, *p* = 0.006)

SBP, systolic blood pressure; NIHSS, National Institutes of Health Stroke Scale; NCCT, non-contrast computed tomography; WBC, white blood cell; HB, hemoglobin; ALB, serum albumin; APTT, activated partial thromboplastin time; TT, thrombin time; LDL, low-density lipoprotein; OR, odds ratio.

## Discussion

sICH is a severe disease with a 30-day mortality rate of approximately 40%, half of these deaths occurring within the first 24 h (16). Early HE is closely associated with a poor prognosis, making early predictions of HE and the development of intervention strategies targeting HE crucial for improving patient survival rates and quality of life (17, 18). Recently, the CT attenuation of intracerebral hematomas on NCCT has recently become a focus and can be used to predict HE, which significantly affects patient outcomes. Studies indicate that the differences in attenuation greater than 18 HU between high- and low-density areas at the edges of a hematoma (19–22) and a minimum attenuation value of ≤31 HU are independent risk factors for HE (23, 24). The HU values of non-low-density areas of the hematoma can also predict HE. However, other studies have reported that the baseline average HU of a hematoma cannot predict HE or poor outcomes (25). One reason for these contradictory findings is that different hospitals use different CT machines; furthermore, even the same patient can have different degrees of CT attenuation across scans. This variability limits the widespread clinical application of hematoma HU values. To overcome this variability, establishing a reference point is necessary. Because the CT attenuation value of normal brain tissue contralateral to the hematoma can serve as an excellent baseline. Therefore, we propose calculating the relative CT attenuation value of the hematoma to predict HE. A method that allows rapid clinical calculation of hematoma CT attenuation values is needed.

The relative CT attenuation is calculated as the difference between the CT attenuation on the hematoma side and that on the opposite side, which reflects the hematoma density. This method can reduce errors arising from differences in CT scanners, acquisition protocols, and segmentation techniques. Our research revealed that the relative CT attenuation value of a hematoma can independently predict HE. While several methods exist for predicting HE using hematoma density or CT attenuation values. For instance, researchers used visual inspection to classify different densities within hematomas and found that low-density areas can independently predict HE (OR values range from 2.65 to 3.42). However, this method relies on subjective visual assessment, which introduces strong heterogeneity and is not conducive to clinical application (7, 26). Alternatively, Jeong et al. (27) used a semiautomatic method to segment hematomas and measure their average HU, finding that CT attenuation in patients with HE was lower than in non-HE patients (55.7 ± 2.9 HU vs. 58.6 ± 3.1 HU, *p*-value < 0.01). Multivariate logistic regression revealed that the average HU of the hematoma was negatively correlated with HE (adjusted OR = 0.64; 95% CI (0.51–0.80)). Xia et al. (25) employed a deep convolutional neural network to automate the measurement of hematoma attenuation values. They predicted adverse outcomes by calculating the difference in average HU between follow-up and baseline CT scans of the hematoma, thereby providing a precise method for quantifying hematoma HUs. Despite their precision, both semiautomatic and fully automatic methods for calculating hematoma HUs require specialized software and exporting patient DICOM images, which is not feasible in most busy hospital settings. In contrast, we used a simple PACS with a built-in circular area tool to quickly determine the relative CT attenuation value within the largest cross-section of the hematoma, facilitating rapid assessment of whether a patient would experience HE. This single-slice ROI approach is particularly useful for patients lacking distinct morphological NCCT markers (e.g., the black hole sign, blend sign, swirl sign, island sign, fluid level sign, etc.). While our method’s accuracy in calculating relative CT attenuation values does not match that of semiautomatic or fully automatic methods, it offers the advantages of speed and accessibility. Numerous previous studies also employ ROI methods based on single layers or limited sections, and also report the independent predictive value for HE and show practical reference values under clinical conditions (22–24). Our study revealed that the OR for hematoma-related CT attenuation was 0.920 (95% confidence interval 0.87–0.98, *p* = 0.006). We also found that a hematoma-related CT attenuation value of ≤33.35 HU predicted HE with an AUC, sensitivity, specificity, positive predictive value, negative predictive value, and accuracy of 0.647 (95% CI: 0.589–0.705), 40.9, 83.1, 59.3, 70.0, and 67.2%, respectively, further validating the feasibility of our method. Our multivariable regression analysis revealed that, in addition to the relative CT attenuation value of the hematoma, the baseline NCCT timing and WBC count were negatively correlated with HE, whereas a history of previous cerebral infarction; the NIHSS score; and the HB, ALB, and TT levels were positively correlated with HE, consistent with previous research findings (28).

Our results indicate an inverse association between the relative CT attenuation value of a hematoma and HE; the lower the relative CT attenuation, the greater the risk of HE. On NCCT scans, high density indicates ICH, and the density of the hematoma dynamically changes in the early stages of the disease due to variations in the proportions of hemoglobin, blood cells, plasma, and other components (29). Initially, early hematomas consist of heterogeneous clumps formed by a mixture of various blood cells and platelet thrombi in protein-rich plasma (30). As platelets aggregate, fibrin network forms, cellular components settle, and plasma components decrease. Clot contraction occurs within the first few hours, leading to the formation of higher attenuation hematomas, which appear on CT images as increased Hounsfield units (HUs) (31, 32). Therefore, the lower the density of the low-density area of the hematoma is, the greater the likelihood of unclotted blood and the higher the risk of HE. Thus, our study provides some basis for the aforementioned mechanisms. The relative decrease reflects the pathological physiological basis of the instability of blood clots: In the acute phase of sICH, the dynamic changes in the density (HU value) of the hematoma are due to the component evolution during blood clot formation. In the early stage of the hematoma, it is composed of heterogeneous blood clots consisting of blood cells, platelet thrombi, and protein-rich plasma; as the coagulation process progresses, platelet aggregation, formation of fibrin networks, deposition of cellular components, reduction of plasma, and blood clot contraction lead to an increase in the HU value. If the blood clot contraction is incomplete or there is persistent active bleeding, the local HU value is low, indicating an unstable blood clot and a higher risk of HE.

We believe that the relative CT attenuation value of hematomas holds significant clinical utility and potential for future applications. First, it can be rapidly determined using standard PACS software without the need for specialized training, additional software, or exportation of DICOM data. This approach also mitigates technical variability across different CT machines, facilitating widespread clinical adoption. Second, unlike qualitative imaging markers, such as mixed signs, swirl signs, and fluid-level signs, the relative CT attenuation value is a quantitative indicator. This quantitative measure can be directly integrated with other numerically expressed predictors (such as laboratory tests and clinical features) to establish specific mathematical models that better predict HE and adverse outcomes. Finally, the relative CT attenuation value can also be used to inform the formulation of ICH treatment strategies. Similar to the spot sign, further research should investigate whether patients with ICH with a relative CT attenuation value of ≤ 33.35 HU would benefit from intensified antihypertensive or hemostatic treatments.

Hematoma attenuation is known to change dynamically in the early stage after symptom onset. This study showed that the time from onset to NCCT was inversely associated with HE in the multivariate logistic regression model (*p* = 0.029). The core rationale for proposing the relative CT attenuation value in this study is to minimize errors due to variations in absolute HU values across different scanning time points. Using contralateral normal brain tissue on the same slice as an internal reference partially corrects differences in the hematoma’s absolute HU values introduced by varying disease progression timelines. Future research should investigate the predictive performance of the relative CT attenuation value across different time windows (e.g., <3 h, 3–6 h, and >6 h).

Using a relative CT attenuation value of ≤ 33.35 HU as the predictive threshold yielded a sensitivity of 40.9% and a specificity of 83.1%. The AUC of the relative CT attenuation value was 0.665, which indicates moderate predictive efficacy. The relatively low AUC and sensitivity of this indicator suggest that hematoma expansion is a complex event determined by multiple factors, and a single imaging parameter is difficult to simultaneously achieve high sensitivity and high specificity. The relative CT attenuation value threshold established by this study should be used in combination with other NCCT markers and clinical features in clinical practice.

This study has several limitations. First, this was a retrospective analysis and had a relatively small sample size. While we determined that the relative CT attenuation value can predict HE, the optimal HU threshold might be different if the sample size were increased. Second, the inclusion of patients qualifying for minimally invasive surgery resulted in a cohort with larger baseline hematoma volumes compared with previous studies. This introduces potential selection bias, partially reducing the reliability of the results. The conclusions need further validation in other cohorts or prospective cohorts. Third, the single-slice ROI approach may fail to fully capture the three-dimensional heterogeneity of hematomas; future studies should explore multi-layer ROIs or 3D segmentation methods to enhance measurement accuracy. Fourth, all enrolled patients underwent baseline CT scans within 24 h of symptom onset, typically before significant contralateral displacement occurred due to severe mass effects. This represents a potential limitation of the methodology; future studies must enhance measurement consistency by establishing standardized operating procedures (specifying precise anatomical locations and exclusion zones for the contralateral ROI). Fifth, as a relative CT attenuation values are positioned as auxiliary predictive indicators rather than independent screening tools and may exhibit low sensitivity and a high potential false-negative rate; therefore, they should be used in conjunction with other NCCT biomarkers and clinical features. Lastly, the operation specifications of the PACS circular ROI tool used in this study were fixed (selecting the largest cross-section and covering most of the hematoma with a circle). Theoretically, the differences among operators were relatively limited, but the study does not report interobserver or intraobserver variability. Given that ROI-based measurements are operator-dependent, assessment of reproducibility is necessary to ensure the robustness of the method in further studies.

## Conclusion

Our data indicate that the relative CT attenuation value of a hematoma can independently predict HE in patients with sICH. But the conclusions of this study warrant cautious extrapolation for patients with small hematomas. The relative CT attenuation value of hematomas may be of greater clinical utility when combined with other NCCT markers and clinical features in clinical practice due to its relatively low sensitivity. Further studies should enroll more samples, explore multi-layer ROIs or 3D segmentation methods, enhance measurement consistency by establishing standardized operating procedures, and report interobserver and intraobserver variability to fully validate these findings.

## Data Availability

The original contributions presented in the study are included in the article/supplementary material, further inquiries can be directed to the corresponding authors.
